# Advanced HIV disease during the first six months on antiretroviral therapy in Zambia: research protocol for a prospective, observational, multi-cohort study

**DOI:** 10.12688/gatesopenres.16359.1

**Published:** 2025-08-27

**Authors:** Thandiwe Ngoma, Aniset Kamanga, Nancy Scott, Allison Morgan, Anushka Reddy Marri, Taurai Makwalu, Lloyd Mulenga, Suilanji Sivile, Mariet Benade, Prudence Haimbe, Hilda Shakwelele, Sydney Rosen

**Affiliations:** 1 Clinton Health Access Initiative, Lusaka, Zambia; 2 Boston University School of Public Health, Boston, MA, 02118, USA; 3 Ministry of Health, Zambia, Lusaka, Zambia; 4 University of the Witwatersrand Johannesburg Faculty of Health Sciences, Johannesburg, Gauteng, South Africa; 5 University of Amsterdam Institute for Global Health and Development, Amsterdam, The Netherlands

**Keywords:** HIV, Zambia, advanced HIV disease, antiretroviral therapy, models of care, cohort study

## Abstract

**Background:**

The proportion of HIV-positive individuals who present for initiation or re-initiation of antiretroviral therapy (ART) with advanced HIV disease (AHD) and are at risk for morbidity and mortality remains high throughout sub-Saharan Africa. In Zambia, where 20% of ART initiators are diagnosed with AHD, little is known about the characteristics of those starting ART with AHD, why treatment initiation is delayed, how AHD clinical management influences clinical and non-clinical outcomes, or implementation of national AHD guidelines at facility level.

**Protocol:**

AHD-Zambia is a mixed-methods observational study to describe AHD clients and care during the first six months after starting or re-starting ART in Zambia. The study will be conducted at 24 public sector primary health facilities in four provinces. It will enroll ART clients screened for AHD during a three-month data collection period (Cohort 1), clients screened for AHD in the 12 months prior to the data collection period (Cohort 2), patients hospitalized for AHD-related conditions (Cohort 3); and clinical providers at the study sites who manage clients with AHD (Cohort 4). Data collection will include quantitative surveys, medical record review during the 12 months before and after enrollment, qualitative interviews, and focus group discussions. Facility-level indicators will also be collected. Outcomes will include detailed profiles of AHD clients and their 6 and 12-month retention in care and viral suppression, provider and client views on barriers to and preferences for AHD care, and assessment of facility fidelity to AHD guidelines.

**Discussion:**

This study will generate a comprehensive profile of clients presenting with AHD in Zambia, including clinical, demographic, social, and behavioral characteristics, treatment outcomes, and barriers to providing guideline-compliant care. Findings will provide insight into the delivery of AHD services, identify gaps in implementation, and support improvements to retention and care during the early treatment period.

**Registration:**

Clinicaltrials.gov NCT06904456.

## Introduction

While many countries have made significant progress toward achieving the UNAIDS 95-95-95 targets for HIV care, ^1^ the proportion of people initiating or re-initiating antiretroviral therapy (ART) with advanced HIV disease (AHD) remains high. ^2^ For adults living with HIV, the World Health Organization (WHO) defines AHD as a CD4 count below 200 cells/mm ^3^ or WHO clinical stage 3 or 4. ^3^ In sub-Saharan Africa, more than 30% of clients initiating ART present with CD4 counts <200 cells/mm ^3^. ^4^ Tuberculosis, cryptococcal meningitis, Kaposi’s sarcoma, and other severe bacterial infections are the leading causes of morbidity and mortality among individuals with HIV. ^5, 6^ A 2023 Lancet HIV commentary noted that, “About a third of adult people living with HIV globally presenting or returning to care have advanced HIV disease, and the estimated mortality rate ratio at ART clinics, compared with people with HIV with CD4 counts greater than 350 cells per μL, has been reported as 3.43”. ^7^ While routine mortality data remain limited, most HIV-related deaths are believed to occur among clients with AHD. ^8– 11^ A CD4 count nadir below 200 cells/μL, moreover, is associated with a poorer prognosis on treatment and more rapid CD4 cell count loss after an interruption to treatment. ^12– 14^

In Zambia, a high-HIV prevalence country in southern Africa, the Ministry of Health (MOH) estimates that approximately 20% of clients initiating or re-initiating ART meet the criteria for AHD. ^15^ Many of these are believed to be clients who are restarting ART after a treatment interruption. ^16, 17^ To address AHD, the MOH issued the Zambia Guidelines for Management of Advanced HIV Disease in 2021. ^18^ The guidelines are tailored to each facility level (community post/health center/clinic/hospital) and includes guidance for screening, diagnosis, and treatment. The Zambia AHD Implementation Framework 2022-2026 ^15^ recommends that clients newly diagnosed with HIV, those re-initiating care, those with high viral load, and those with severe illness be prioritized for AHD screening. The framework also recommends screening using WHO staging or CD4 testing and advises on additional laboratory screenings and prophylaxis and treatment to prevent/treat comorbid conditions.

Although the AHD guidelines and implementation framework are comprehensive, implementation still varies widely in practice. ^19^ Facilities with stronger infrastructure tend to offer more complete AHD care, while under-resourced sites may lack the diagnostic tools, medications, or trained staff needed to deliver the package. In addition, limitations to the national electronic medical record system, SmartCare, ^20^ make it difficult to evaluate implementation fidelity or track outcomes without primary data collection. Smartcare neither routinely capture clients’ AHD status or specialized care provision for AHD, nor does it readily allow for cross-facility tracing. The absence of cross-facility tracing limits the ability to assess return to care among clients who silently transfer or disengage and then return to care, many of whom are at risk for AHD. ^16, 17^

Beyond service delivery challenges, little is known about why individuals in Zambia do not start or re-start treatment before progressing to AHD, how the care they receive influences clinical and non-clinical outcomes, or how they transition to a non-AHD or “post-AHD” status. Data on social and behavioral characteristics of clients with AHD, beyond age and sex, are limited. Factors such as employment demands, stigma, or past experiences with the healthcare system may delay care-seeking until symptoms become severe. ^21^ Similarly, clients from key or marginalized populations, such as commercial sex workers, men who have sex with men, or survivors of intimate partner violence, may face additional barriers to early engagement in HIV care and therefore be at risk of developing AHD. ^22^

The Advanced HIV Disease During the First Six Months on Antiretroviral Therapy in Zambia Study (AHD-Zambia) will use mixed methods to describe the characteristics, experiences, and outcomes of clients diagnosed with AHD at ART initiation or re-initiation. Focusing on the early treatment period (first six months after initiation or re-initiation), the study will collect data on clinical and socioeconomic characteristics, HIV care history and experiences, clinical outcomes, and provider perspectives to understand how clients with AHD are identified, managed, and retained in care. The study’s overall goal is to generate information to better understand who is presenting with AHD in Zambia, how they are currently managed by the healthcare system, and their treatment outcomes after starting or restarting ART. Findings from this study will help inform improvements to Zambia’s national HIV program and identify opportunities to strengthen AHD care during the early treatment period.

## Protocol

**Figure 1.**
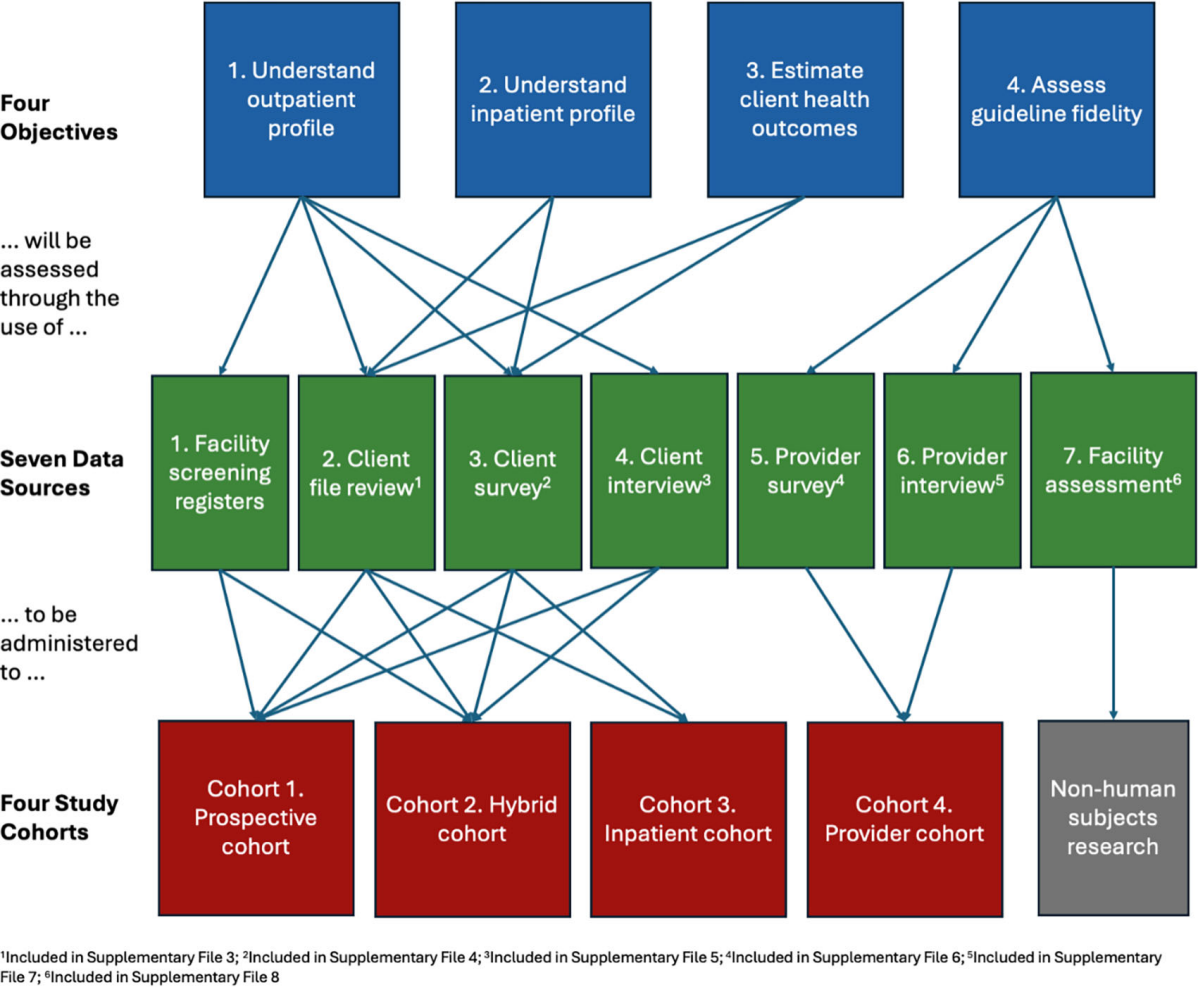
AHD Zambia study objectives.

AHD-Zambia is a mixed-methods, multi-cohort observational study designed to generate a detailed picture of AHD care-seeking and service delivery during clients’ first six months on ART. The study will collect both retrospective and prospective data to evaluate outcomes of clients with AHD and assess how providers and the health system manage AHD care at 24 health facilities in Zambia. The study includes three discrete patient cohorts and one provider cohort (described below); facility-level capacity assessments; and supplemental electronic medical record (EMR) data from the national SmartCare system. The full study protocol is provided as Supplementary file 1; EMR fields to be collected are listed in Supplementary file 2. The overall structure of the study is illustrated in Figure 1.

This study is guided by the RE-AIM framework to assess five key implementation science domains: Reach, Effectiveness, Adoption, Implementation, and Maintenance. ^23^ In parallel, we will also apply the COM-B model of behavior change to explore the mechanisms underlying challenges that may be relevant to our study population. Specifically, we will explore care-seeking behavior among treatment clients and guideline implementation behaviors among treatment providers. The COM-B model posits that behavior is influenced by three core components: *Capability* describes the physical (e.g. physical health, strength) and psychological (e.g. knowledge, cognitive skills, etc.) capacities required to carry out a behavior. *Opportunity* refers to external factors that enable or constrain a behavior including physical access (e.g. access to the clinic for clients, equipment available for providers) and social influences (e.g. role of others’ opinions, stigma, professional culture). *Motivation* includes both reflective processes (e.g. beliefs about capabilities, consequences, etc.) and automatic processes (e.g. emotions, impulses, habits). Integrating both RE-AIM and COM-B, allows us to assess both the implementation outcomes and the behavioral drivers shaping them, giving us a better understanding of how and why AHD support succeeds or can be strengthened in routine settings.

### Objectives

AHD-Zambia has four primary objectives. First, we will create a comprehensive profile of clients with AHD presenting for ART initiation or re-initiation at outpatient healthcare facilities in Zambia. Second, we will describe the profile and referral pathways of clients admitted to hospitals for AHD. Third, we will estimate the treatment outcomes of clients presenting with AHD, focusing on retention in care and viral suppression, and identify predictors of those outcomes. Finally, we will assess fidelity to Zambia’s AHD guidelines and care package at the facility and provider levels and describe resource utilization.

### Study population and cohorts

We will recruit participants into four discrete cohorts during a 3-5 month data collection period in mid 2025 (anticipated May-August 2025). Each cohort is described in detail in Table 1. All study participants will be adult (≥18) HIV clients or HIV care providers at the study sites. All participants must be able to communicate in one of the languages used in the study (English, Bemba, Nyanja, and/or Tonga). Only clients who are not currently on ART or have been on ART for six months or less at the time of AHD screening are eligible for enrollment. ART initiators may include both ART-naïve individuals and ART-experienced clients restarting treatment. Eligible providers for the fourth cohort include up to five healthcare workers directly involved in the delivery of AHD care at each study site.

**Table 1.** Study cohorts.

Cohort name	Description and purpose	Inclusion criteria	Exclusion criteria	Data to be collected	Anticipated sample size	Comments
Cohort 1: Prospective cohort	Clients starting or re-starting ART who are enrolled in the study on the day of AHD screening or immediately after and who can be followed prospectively from the point of AHD diagnosis, to minimize recall bias.	1) On ART for ≤6 months since most recent treatment initiation; 2) Screened by the study clinics for AHD at ART initiation or at their first follow-up visit within one month of initiation (delay is allowed for clients whose physical condition may not allow study enrollment on the day of AHD screening).	1) Pregnant and/or receiving antenatal care; 2) Too ill at the time of AHD screening and at the first follow up visit to participate in the study.	• Client quantitative survey ^1^ on day of study enrollment; • Medical record data ^2^ for up to 12 months before and after AHD screening; • If consent for re-contact, individual in-depth interviews (IDI) ^3^ or focus group discussion (FGD) within 12 months of study enrollment.	2,232	Participants referred from a study site to inpatient care will be followed up at the inpatient facility if feasible but will remain in Cohort 1 for analysis. Participants screened for AHD but found not to meet the definition of AHD (not diagnosed with AHD) will be retained in Cohort 1 to serve as a comparison group.
Cohort 2: Hybrid cohort	Early ART clients screened for AHD in the year before the start of the data collection period who can be followed retrospectively through medical record review and participate in prospective data collection if they return to the study sites during the study enrollment period.	1) Screened for AHD ≤12 months prior to start of data collection period, as indicated by each site’s AHD register; 2) On ART for ≤6 months at time of AHD screening.	1) Pregnant and/or receiving antenatal care; 2) For those who return to care, >6 months on ART at time of return visit; 3) Too ill to participate in study at time of return visit (for those who return to care).	• Medical record data ^2^ for up to 12 months before and after AHD screening; • For those who return for care during the data collection period and provide consent, client quantitative survey ^1^, IDIs ^3^, and FGDs.	8,928	Cohort 2 will allow a much larger sample size of current or recent clients with a AHD than we anticipate being able to enroll in Cohort 1 alone.
Cohort 3: Inpatient cohort	Clients hospitalized for AHD-related reasons, to develop a profile of AHD inpatients and observe outcomes after hospital discharge.	1) Admitted to inpatient care for AHD-related illness; 2) On ART for ≤6 months at hospital admission.	1) Pregnant and/or receiving antenatal care; 2) Not physically, mentally, or emotionally able to participate in the study prior to discharge; 3) Confined to tuberculosis isolation ward, intensive care unit, or other ward specifically for clients with acute infectious disease.	• Medical record data ^2^ for up to 12 months after AHD screening; • Baseline survey ^1^ prior to hospital discharge.	360	Participants will be followed to the referring primary care clinic for EMR data where possible.
Cohort 4: Provider cohort	Study site clinical providers involved in AHD care to learn more about AHD-related service delivery, decision-making, and guideline fidelity.	*Inclusion*: 1) Employed by or at the study site for at least 6 months; 2) Directly interact with clients presenting with AHD.	None.	• Provider survey ^4^ with closed- and open-ended questions; • Qualitative IDIs ^5^.	120	We will enroll up to 5 providers at each study site.

^1^ Baseline survey is included as Supplementary file 4.

^2^ Fields to be collected from medical record data are described in Supplementary Files 2 and 3.

^3^ Client IDI guide is included as Supplementary file 5.

^4^ Provider survey is included as Supplementary file 6.

^5^ Provider IDI guide is included as Supplementary file 7.

Clients will be eligible for Cohort 1 or 2 if they are offered AHD screening by clinic staff and meet other study criteria as shown in Table 1. Cohort 3 will be comprised of clients who have been hospitalized for conditions or complications related to AHD. We anticipate that each facility will have its own procedures for determining whom to screen for AHD and have access to different resources for AHD screening (e.g. some will have CD4 count technology on site, while others will have to send samples to external laboratories for CD4 counts). As this is a purely observational study and we do not have resources to conduct study-specific tests for AHD, we chose to rely on routine clinic procedures to identify eligible participants, which in turn will make our study samples an accurate reflection of actual practice. Participants who are classified by the study clinics as initiating or re-initiating treatment and are enrolled in the study but are later discovered (through study data collection) to have transferred to the study site as silent transfers will be retained in the study.

### Study sites

**Figure 2.**
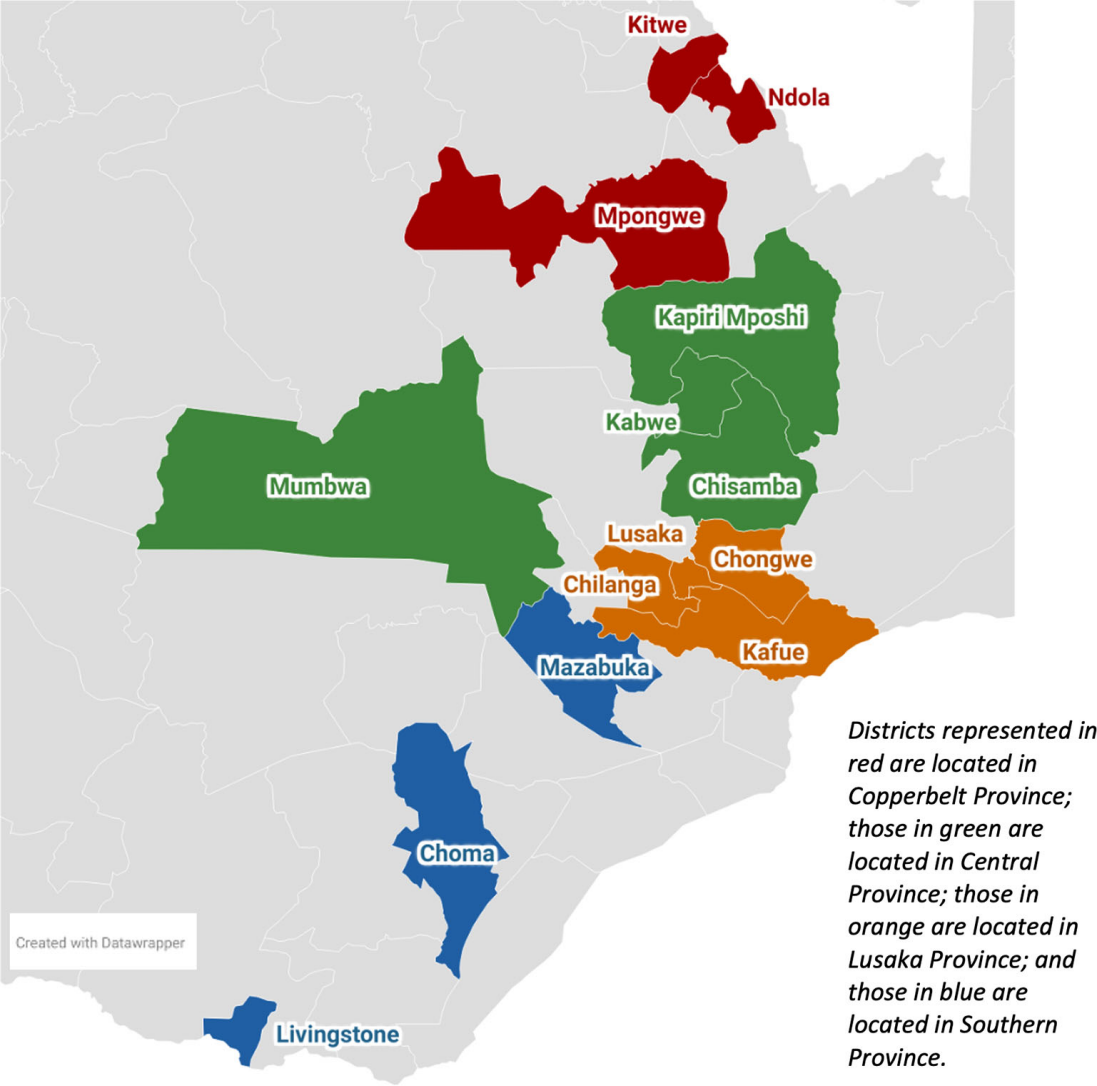
Districts in which study sites are located.

The study is being conducted at 24 healthcare facilities in Zambia’s Lusaka, Central, Copperbelt, and Southern provinces ( Figure 2, Supplementary Table 1). Of these 24, the 12 in Central and Lusaka provinces have previously served as sites for the AMBIT Project’s SENTINEL study ^24^ and the Retain6 PREFER survey. ^25^ The 12 in Copperbelt and Southern provinces were selected at the request of, and in consultation with, the Ministry of Health to comprise a more nationally representative sample in high HIV prevalence areas. Sites were chosen to capture variation in setting (urban and rural), facility size, HIV burden, and level of support from implementing partners. All study sites provide HIV treatment services and use the national electronic medical record system, SmartCare.

Zambia has implemented a “hub-and-spoke” system for management of AHD and coordination between health facilities. ^15, 26^ Spokes, which are lower-level facilities including health posts, health centers, and local clinics, offer outpatient services and basic routine care for AHD clients. Spokes refer clients to hubs, which are first level hospitals and referral hospitals for inpatient care or advanced services for serious conditions. Eight of the 24 AHD-Zambia study sites are hubs, offering inpatient care as well as outpatient services; the remaining 16 are spokes and must refer clients to the nearest hub for hospital admission.

### Recruitment, study eligibility screening, informed consent, and data collection

Procedures for recruitment of study participants, screening for study eligibility, informed consent, and data collection are shown in Table 2.

**Table 2.** Study procedures.

Cohort name	Recruitment	Screening for study eligibility	Informed consent	Data collection
Cohort 1: Prospective cohort	Potential participants will be recruited sequentially as they are screened for AHD and entered in the AHD register. Clinic staff will be asked to refer potentially eligible clients to the study assistant located on site.	A study assistant will complete a screening form to confirm potential participants’ eligibility. Clients who are too ill to provide consent on the day of AHD screening will be re-approached at their next clinic visit if it occurs within one month of initial AHD screening. Clients who are eligible for the study but are immediately referred for inpatient care (at the study site or elsewhere) will be included in Cohort 1.	Study eligible clients will be asked for written informed consent to participate. Primary consent will include the baseline survey and medical record review. An additional, optional consent will allow re-contact for potential participation in IDI or FGD. (For those who are recontacted and agree to participate in the qualitative data collection, an additional consent for the IDI or FGD and audio-recording will be obtained at that time.)	Upon consent, participants will be administered the AHD-Zambia client survey. We will also collect data from participants’ electronic and paper medical records for the period from 12 months before to 12 months after AHD screening. Participants with confirmed AHD may participate in qualitative individual interviews or focus group discussions.
Cohort 2: Hybrid cohort	We will use the paper registers kept by the study sites for recording those screened for AHD to identify all eligible clients. A list of Cohort 2 participants will be given to each site’s entry clerk so that clients from this cohort who visit the site during the data collection period can be referred to the study assistant for screening and consent for additional data collection.	No additional screening for eligibility is required for medical record review; all adults identified during recruitment (AHD register review) will be eligible. Participants in Cohort 2 who return to the site for a visit during the data collection period will be screened for eligibility for additional data collection.	No consent will be sought from Cohort 2 for medical record review. The study will have no interaction with Cohort 2 participants who do not return for a clinic visit during the study period. Participants in Cohort 2 who return to the site for a visit during the data collection period and are referred to the study assistant will be asked for written informed consent (same terms as Cohort 1).	Electronic and paper medical records will be reviewed, as described below. Those who return and consent will complete the AHD-Zambia client survey and may be invited to participate in IDIs and/or FGDs provided they were confirmed to have AHD.
Cohort 3: Inpatient cohort	At the 8 study sites with inpatient wards, clinical staff will be asked to inform the study assistant when a patient is admitted for an AHD-related condition and the study assistant will review the admission register to identify potential participants.	No additional screening for eligibility is required. Hospital staff will inform the study assistant when they believe that an eligible patient is stable, can understand the consent process, and can complete the survey. This may be at any time before discharge or immediately after discharge.	Procedures for administering consent to these respondents will be identical to those for Cohort 1. A caregiver or hospital employee who is not assisting with study enrollment may be present for the consent process at the potential participant’s request.	Upon consent, participants will be administered the AHD-Zambia client survey, with additional questions relevant to inpatient admission and care. To the degree possible, we will also collect data from participants’ electronic and paper medical records for the period from 12 months before to 12 months after AHD screening. Cohort 3 participants who continue their HIV care at non-study facilities will be followed up with EMR data if such data are made available to the study team. If not, the observation period for these individuals will end with hospital discharge.
Cohort 4: Provider cohort	Potential participants in Cohort 4 will be referred to the study team by each site manager, based on the inclusion and exclusion criteria shown in Table 1.	No screening for study eligibility is needed, as only eligible providers will be referred by the site manager.	Healthcare workers referred to the study assistant will be asked for written informed consent prior to their interviews.	Upon consent, the study assistant will administer the AHD-Zambia Cohort 4 survey. No identifiers of any kind will be collected from this cohort. We will record only their professional cadres and number of years of professional experience. Cohort 4 participants will also be invited to participate in qualitative interviews At their convenience, after completion of the quantitative survey, during the three-month data collection period.

As shown in Table 2, medical records will be reviewed for all participants in Cohorts 1-3, with a waiver of consent. Fields to be collected from electronic medical records in Smartcare, listed in Supplementary file 2, cover participants’ demographic characteristics and a comprehensive set of HIV treatment indicators are. Where data are missing from SmartCare and for fields not available in SmartCare, we will also review participants’ paper clinic files and other site-level registers that may contain relevant data.

Participants in Cohort 1 and Cohort 3 and those in Cohort 2 who return for a visit during the data collection period will be administered the AHD-Zambia client survey (Supplementary file 3). Cohort 3 participants will also complete a module about the inpatient experience. For all Cohort 1-3 participants, the survey will be administered in a private room or a ward space with sufficient privacy to ensure confidentiality of responses. Providers in Cohort 4 will complete the AHD-Zambia Provider survey (Supplementary file 4).

For Cohort 4, the survey and interview guides describe AHD management practices, framed around providers’ capability and opportunities to deliver AHD care and motivations for guideline compliance. The survey will ask closed-ended questions about respondents’ experience with AHD, understanding of national guidelines, clinic capacity and barriers to providing AHD care. The interviews will ask the same providers qualitatively about how AHD cases are identified and managed within the evolving context of their specific clinic and elicit recommendations for improvements.

**Figure 3.**
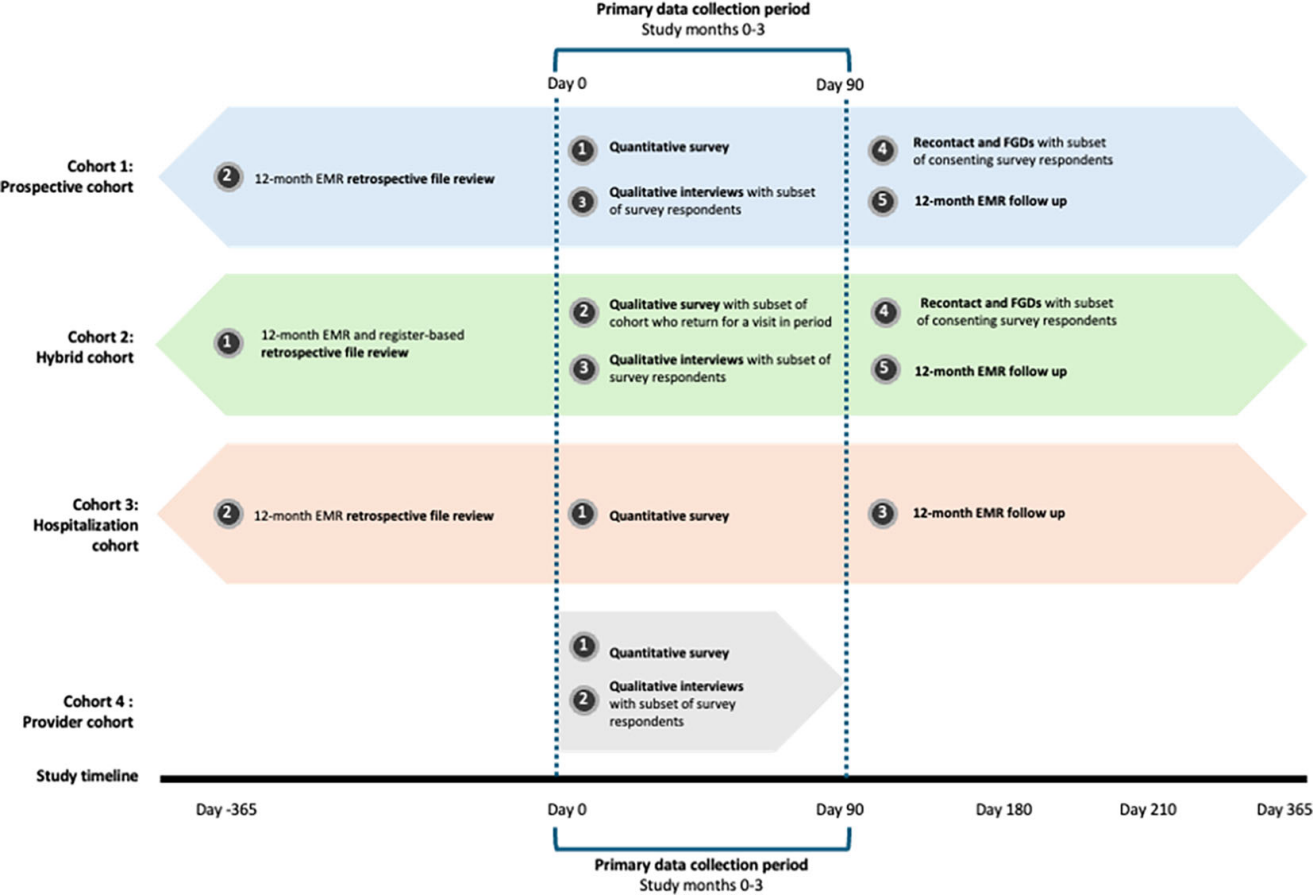
Data collection activities and timeline. Numbers in the dark grey circles indicate the sequence of data collection events. For example, for Cohort 1, we will 1) enroll clients and conduct the quantitative survey; then 2) review EMR data for the 12 months preceding study enrollment; then 3) conduct qualitative interviews with a subset of quantitative survey participants; then 4) recontact consenting participants for participation in a focus group discussion; finally 5) review EMR data for the 12 months following study enrollment.

The sequence of data collection for each cohort is illustrated in Figure 3.

### Facility assessment data

In addition to individual-level data from clients and providers enrolled in our four cohorts, facility-level and aggregate data will be collected to assess structural and operational factors influencing AHD service delivery. A structured observation tool (Supplementary file 8) will be used at each study site to evaluate availability and completeness of clinical registers, presence of national guidelines and job aids, availability of diagnostics and medications, staff numbers and expertise for each relevant cadre, patient and visit numbers, and facility capacity to implement the AHD care package. Aggregate data will also be drawn from the national District Health Information System and national reports from SmartCare. Aggregate and facility assessment data are not considered human subjects data for ethics purposes.

### Data management

AHD-Zambia will collect three different types of human subjects data—survey responses, medical record data, and qualitative data—each with its own management procedures.

Data from surveys (all cohorts) will be entered live at the time of the interview into an electronic database on Survey CTO (Dobility 2023) using encrypted handheld tablets. If there are power or cellular access failures, data will be entered onto paper study forms and then transcribed into a database at the local study office. Data will be stored on Survey CTO’s secure, cloud-based server. Survey data will be converted to SAS, STATA, or R for final cleaning and data analysis.

A study screening log will be maintained by research assistants to document Cohorts (1-3) eligibility screening, the consent process, and survey completion to allow for monitoring of potential biases due to differential consent. This log will not contain personal identifiers and will collect limited demographic data (age, gender, and ART duration) solely for eligibility determination.

Participant surveys will be assigned an 8-digit, sequential identification number (study ID). The study ID number will be used to identify individual participants in the study databases and for all data analysis. Identifiers needed to link individuals across data sources (e.g. surveys, SmartCare and paper medical records and logs), which will include name, date of birth, and client identification numbers, will be collected separately from survey responses in a secure file with an encryption key in Survey CTO. Identifying information will be used solely for the purpose of linking data for individual participants. All databases will be password protected with access restricted to relevant members of the study team.

The provider survey and interview will be administered to all respondents in Cohort 4. Respondents will be assigned a study ID, and job titles, years in position, and other descriptive information will be recorded, but we will not collect dates of birth, ID numbers, or other identifiers.

Qualitative responses, including IDI and FGD recordings, will be transcribed verbatim and translated into English where applicable. Transcripts will be anonymized prior to analysis. A de-identified version of the study dataset may be made publicly available in a data repository in accordance with funder requirements and institutional policies, ensuring that no individual participant can be identified.

### Data analysis

We will begin by creating descriptive summaries of the demographic, behavioral, and/or clinical characteristics of study participants in each cohort using frequencies and proportions for categorical variables and means with standard deviations or medians with interquartile ranges for continuous variables. Client survey responses will be stratified by time on ART, ART history (naïve vs. non-naïve), age, sex, and/or site characteristics such as province, facility size, and setting (urban vs. rural). Provider responses will be stratified by professional cadre.

For Cohorts 1, 2, and 3, clinical outcomes will be assessed utilizing follow-up data collected from participants’ medical records. We will conduct a crude analysis reporting simple proportions with 95% confidence intervals of patients achieving the retention outcome, defined as not missing a scheduled clinical or medication pickup visit during the first 6 months after treatment initiation by more than 28 days. Next, we will compare the proportions of patient disengaged from care by key variables including age, gender, baseline CD4 cell counts, baseline viral load results, clinical stage at ART initiation, presence of opportunistic infections, site characteristics, time on ART and naïve v non-naïve treatment status. Risk ratios and risk differences, along with 95% confidence intervals, will be estimated using logistic, log-binomial or log-linear regression models. If appropriate, models will be adjusted for covariates associated with the outcome and/or exposure. In cases where effect measure modification is detected, we will present stratified results.

For Cohort 1 and participants in Cohort 2 who return for a visit and are consented during the data collection period, we will compare characteristics between participants with confirmed AHD and those who were screened for AHD but found not to meet AHD criteria. The analysis will include a simple comparison of the groups with respect to baseline predictors of outcomes to look for any imbalances. These potential confounders include demographic and clinical variables and geographic and facility-level factors. We will then conduct a crude analysis comparing the proportion of patients achieving the dichotomous retention outcome by group. Using a log-linear regression model, we will estimate crude risk ratios and crude risk differences and their corresponding 95% confidence intervals. Should any important imbalances be observed, we will proceed with an adjusted model. In cases where effect measure modification is detected, we will present stratified results.

Qualitative data from IDIs and FGDs will be audio recorded, transcribed, and translated into English as necessary. Interview transcripts will be analyzed in NVivo using the Framework Method. ^27^ We will apply a hybrid inductive–deductive coding approach, with core themes aligned to interview guides and implementation science frameworks such as COM-B ^28^ and RE-AIM. ^23^ Additional codes will be developed based on emerging content during transcript review. Data will be summarized into matrices, and illustrative quotations will be used to highlight key findings related to AHD care delivery, provider decision-making, and patient experiences.

We will analyze facility-level data by creating descriptive summaries of the characteristics and capacity of each clinic using frequencies and proportions for categorical variables. We will create a guideline adherence score for each ste core component of the AHD guidelines. Each step will be scored as complete, partially complete, or not completed. Individual components and the overall score (proportion of applicable steps completed) will be calculated as a proportion of overall steps, then categorized as low, medium or high.

### Data integration and interpretation

We will use a convergent mixed-methods design to integrate quantitative and qualitative data across patient, provider, and facility levels. Each data source will be analyzed independently using the methods described above, then integrated at the interpretation phase using joint display matrices ^29, 30^ to map finding across the RE-AIM domains and COM-B constructs. This will enable the identification of convergence (e.g. quantitative gaps explained by qualitative barriers), complementarity, and seemingly divergent findings. Divergent findings will be examined using theory-driven interpretation, ^29^ leveraging the COM-B model and RE-AIM domains to identify whether observed gaps may be attributable to limitations in individual capability, external opportunity, motivational alignment, or broader implementation challenges. Data will be presented by facility and overall.

### Sample size

The true burden of AHD and frequency of screening for AHD in Zambia are unknown. This exploratory, minimal-risk study will therefore aim to enroll as many respondents as possible to get a clear picture of AHD care provision. We anticipate having the resources and time to enroll at each site for a period of 3 months, with ultimate enrollment numbers determined by frequency of eligible patients’ clinic visits and study assistant capacity. Based on historical patient volumes, we expect that Cohort 1 will have a maximum enrollment of 2,232 participants, Cohort 2, 8,928 participants, Cohort 3, 360 participants, and Cohort 4, 120 participants. Our overall sample size for the study will thus be 11,640 participants, rounded up to 11,700 in the approved protocol. Details on sample size estimates for each cohort are included in the study protocol (Supplementary file 2).

### Dissemination of results

The study results will be disseminated in several ways. The primary audience for study findings is the Zambian Ministry of Health and its partners, who may use the results to inform programmatic improvements in AHD care and the early treatment period. Broader dissemination will include peer-reviewed publications, presentations at national and international conferences, and policy briefs. Only aggregate and stratified data will be presented to protect confidentiality; no individual participants will be identifiable in any dissemination products.

### Limitations

We anticipate that AHD-Zambia will have a number of limitations. Most important, we can only enroll participants who actively present at the study facilities and, for Cohorts 1 and 3, are not too ill to consent for study enrollment. The study will not capture the characteristics and experiences of individuals with AHD who never seek care, are not able to consent, and/or die outside the healthcare system. Generalizability will be limited by the number of study sites (24 sites across four provinces, out of a total of approximately 3500 healthcare facilities in Zambia’s ten provinces), the requirement that study sites have access to SmartCare, and the sample size and relatively brief follow up period, both of which are constrained by study resources and data access. For some components of data collection, survey participants will provide responses about their experiences up to 6 months after AHD screening, creating the possibility of recall bias. Survey and qualitative data will depend on the accuracy of participant self-report, which cannot be verified and may reflect some degree of acceptability bias. we will utilize routinely collected. EMR data, for which we will rely for clinical outcomes and service provision data, are known to be incomplete, with many missing fields, and, more important, do not capture clients accessing care at other facilities. Silent transfers are common ^31^ and may result in classifying some participants as disengaged when they have in fact continued treatment at a different facility.

### Ethics review

The AHD-Zambia study protocol was approved by the Institutional Review Board at Boston University Medical Campus (BUMC IRB H-45653, approved 9 April 2025), the ERES Converge Institutional Review Board in Zambia (2025-Feb-037, approved 18 March 2025 **)**, and the Zambian National Health Research Authority (NHRA-1973/21/02/2025, approved 10 March 2025). The Zambian Ministry of Health (MOH) participated in protocol development and MOH representatives will serve as co-investigators for the study. All participants will provide written informed consent prior to survey and qualitative data collection. Medical record review will be conducted under a waiver of informed consent, as approved by the relevant ethical review committees. All study procedures adhere to ethical principles outlined in the Declaration of Helsinki. AHD-Zambia is registered at Clinicaltrials.gov as NCT06904456.

### Current status of the study

Medical record data extraction for AHD-Zambia began in April 2025. Enrollment of participants for Cohorts 1-3 is anticipated to take place between May and August 2025. Qualitative data collection and follow up for clinical outcomes will begin in May 2025 and continue through March 2026 or later.

## Discussion

The proportion of adults with HIV in sub-Saharan Africa who present for treatment initiation or re-initiation with advanced HIV disease has remained high and relatively constant despite near universal geographic access to antiretroviral therapy. Improving AHD care is thus a high priority throughout the region. Prerequisites for doing so are better information about implementation of the existing AHD guidelines, an accurate description of those who have AHD, the needs and preferences of AHD patients, and facility capacity and barriers to providing high quality AHD care. The AHD-Zambia study is designed to generate this information to assist the Ministry of Health and others in strengthening AHD care and improving treatment outcomes. It will generate one of the first comprehensive clinical, demographic and behavioral profiles of clients presenting to for routine AHD care at primary level facilities. It will also gather knowledge about the structural factors that influence late presentation for care and client and provider failure to comply with AHD guidelines. Finally, it will reveal variation among healthcare facilities, by size, province, and level, helping to identify factors that support higher quality care and where additional resources are needed.

## Data Availability

No data are associated with this article. Extended data are available from https://hdl.handle.net/2144/50511. ^32^ CC BY-NC 3.0 US Attribution-NonCommercial 3.0 United States. Supplementary Table 1. Study site characteristics Supplementary File 1. AHD-Zambia full protocol Supplementary File 2. Fields to be collected from electronic medical records Supplementary File 3. Client file review instrument Supplementary File 4. Client survey instrument Supplementary File 5. Client interview guide Supplementary File 6. Provider survey instrument Supplementary File 7. Provider interview guide Supplementary File 8. Facility assessment tool Supplementary File 9. Client survey information form Supplementary File 10. SPIRIT checklist
